# Health in the service of state-building in fragile and conflict affected contexts: an additional challenge in the medical-humanitarian environment

**DOI:** 10.1186/s13031-015-0039-4

**Published:** 2015-03-29

**Authors:** Mit Philips, Katharine Derderian

**Affiliations:** Analysis and Advocacy Unit, Médecins Sans Frontières (MSF), Rue de l’Arbre Bénit 46, 1050 Brussels, Belgium

**Keywords:** Health systems, Fragile contexts, Conflict, Humanitarian aid, Access to care, State building, Post 2015 agenda

## Abstract

**Background:**

Global health policy and development aid trends also affect humanitarian health work. Reconstruction, rehabilitation and development initiatives start increasingly earlier after crisis, unleashing tensions between development and humanitarian paradigms. Recently, development aid shows specific interest in contexts affected by conflict and fragility, with increasing expectations for health interventions to demonstrate transformative potential, including towards more resilient health systems as a contribution to state-building agendas.

**Discussion:**

Current drives towards state-building opportunities in health interventions is mainly based on political aspirations, with little conclusive evidence on linking state-building efforts to conflict prevention, neither on transformative effects of health systems support. Moreover, negative consequences are possible in such volatile environments. We explore how to anticipate, discuss and monitor potential negative effects of current state-building approaches on health interventions, including on humanitarian aid.

Overriding health systems approaches might increase tension in fragile and conflict affected contexts, because at odds with goals typically associated with immediate emergency response to populations’ needs. Especially in protracted crisis, quality and timeliness of humanitarian response can be compromised, with strain on impartiality, targeting the most vulnerable, prioritising direct health benefits and most effective strategies.

State-building focus could shift health aid priorities away from sick people and disease. Precedence of state institutions support over immediate, effective health service delivery can reduce population level results.

As consequence people might question health workers’ intention to privilege health above political, ethnic or other alliances, altering health and humanitarian workers’ perception. Particularly in conflict, neither health system nor state are impartial bystanders.

**Summary:**

In spite of scarce evidence on benefits of health systems support for state-building, current dominant line of thought among donors might influence aid strategies and modalities in settings of crisis, conflict and longer-term health system fragility. Negative consequences may arise from dominance of political agendas over health needs, with risk for effectiveness, nature and perception of health interventions. Potential effects in at least three key health areas merit critical review: quality of humanitarian health interventions, tangible contributions to population level health benefits, perception of health and humanitarian workers. To keep health needs as yardstick to determine effective health and humanitarian priority investments, is challenging.

## Background

Global health policy trends in development might affect also humanitarian health work, both due to their influence on the overall health arena humanitarians work in and the potential blurring of the lines in the perception of health actors with different missions. Humanitarian health interventions primarily respond to human suffering and need to impact rapidly and directly by mitigating excess mortality and morbidity. In medical humanitarianism, the medical act itself and its immediate impact on mortality are of value, not its contribution to other wider goals, however lofty. Beyond the medical-humanitarian sphere, other health actors may aspire to contribute to health systems development or wider effects outside the health sector, such as state legitimacy or peace building. This translates into stark differences in operational and programmatic approaches [1,2].

The tension between, and the challenge of linking up, emergency humanitarian and development aid approaches are a long-standing and unresolved debate among analysts and practitioners [3,4]. This article aims to unpack the potential effects of the most recent chapter in the ongoing debate —the aspiration to use health systems support in the service of state-building. We examine the implications of this new approach in conflict or crisis settings, as well as its implementation in post-conflict, fragile or development settings, to explore what limitations arise in health systems support as a transformative instrument beyond the health sector and what stakes for humanitarian and health aid emerge.

At the beginning of the millennium, global health focused on population-based results in reductions of ill health and mortality, as a basis of and precondition for human and economic development; it was at this point in time that the Millennium Development Goals (MDGs) took form, both at global and at country level. Current trends in the health policy and aid debate have shifted from attacking major killer diseases, towards favoring support to existing health systems. The paradigm of health systems is now incorporated in the policy of most health donors, including global health initiatives created to target specific diseases or health interventions, such as the Global Fund for AIDS Tuberculosis and Malaria (GFATM), PEPFAR and GAVI. Consequently, this paradigm shift has influenced the profile and approach of most implementing actors in health.

Recently, development aid has showed specific interest in conflict and fragility, not merely as complicating factors, but as subjects of development interventions per se. Increasingly health interventions now are expected to demonstrate transformative potential, including prominent objectives of peace and state building. Regularly, health interventions limited to humanitarian relief are criticized as a missed opportunity or even as a potential threat for systems development and state building [5,6]. Increasingly, concerns about effectiveness and impartiality of humanitarian health interventions seem to lose ground in favor of the political and development narrative of health for state building.

The current focus of development aid on countries that combine lowest income and least progress towards the MDGs, has brought additional donor attention to crisis- affected and fragile states. The World Bank report has identified violence and conflict as obstacles for development progress and proposes the building of credible state institutions as a mitigating intervention [7]. This type of state building as prevention for repeated cycles of violence –in particular in fragile contexts- is seen as a possibly less expensive alternative to interventions during conflict, such as the deployment of peace keeping forces etc. [8-11]. These preventive, cost-limiting aspects have rekindled interest in the potential of health care as a transformative action. A striking parallel can be seen in the emergence of the concept of resilience in crisis affected populations, its interest possibly reinforced by the economic crisis in donor countries [12-14].

Health interventions used by states and non-state actors to achieve foreign policy objectives is a controversial yet growing part of health diplomacy [15]. Specifically, in post-conflict contexts and situations of protracted crisis, both humanitarian and development actors appear simultaneously, along with agencies with mixed mandates. During the last decade, reconstruction, rehabilitation and development initiatives have started increasingly earlier in the post-crisis phase, increasing tensions between different paradigms.

Following Busan and the Dili declarations [16], the aid effectiveness agenda has developed a specific interest in fragile states. A group of fragile and conflict affected countries, development partners and international organisations announced the ‘New Deal’ for engagement in fragile states. This group has taken on ‘Peace and State building Goals’ as the basis for progress towards the MDGs in fragile contexts, with a focus on country-led and owned transition processes out of fragility [17].

Within the global consultation on the post 2015 development agenda, discussions went well beyond health, into the specific topic of conflict and fragility. The G7+ has requested that peace and state building goals be integrated into the Sustainable Development Goals. Hence, the focus of health in fragile states might be increasingly expanded from a contribution to the MDGs to a tool of conflict prevention and state building per se. A recent proposal to create ‘humanitarian goals’ and the debate it spurred shows its presence in the current policy debate [18,19].

## Discussion

### High expectations of health as broader transformative instrument in a wide variety of contexts

The current drive to emphasize state building opportunities in health and health systems interventions is mainly based on political aspirations and concepts. The jury is still out as regards the evidence for health systems support as a transformative instrument beyond the health sector.

‘Health as a bridge for peace’ is not a new concept. Though it proved difficult to operationalize and apply in the past, it remains appealing and variations on the theme proliferate as peace dividend, violence mitigation or conflict transformation. Even if evidence is scarce on the expected benefits of health for state building [5,20,21], it currently remains the dominant line of thought among donors. For health actors dependent on donor preferences, it is hard to challenge the received wisdom of this policy trend. By and large, many organisations already adopt this donor paradigm, ahead of field based reality checks.

A major difficulty in the current policy discussion is the tendency to lump together ‘fragile and conflict affected contexts’ (FCAS) as one group [22-25], implying that a similar operational and political approach could work across different countries in such a heterogeneous group. This practice contrasts with earlier recommended differentiation according to typology [26-28]. Even without commenting on the varying interpretations of ‘fragility’ by different actors, conflict as ingredient of the mixture changes things fundamentally and renders the proposed ‘peace and state building’ approach rather explosive. The approach proposed in the New Deal treats the role of states in a highly abstract and apolitical manner, eliding states’ own implication as potential actors in violence or partisans within conflict. Recent developments in South Sudan and CAR, both in 2013 piloting ground for several state and peace building initiatives [29], highlight the governments’ implication in new outbreaks of violence with ethnic, religious or political character.

The definition of fragility includes countries *unable* but also *unwilling* to provide essential services to their population [30,31], but in the current paradigm, the latter consideration seems to have been left out of the equation, as donors aim to support existing state institutions and government led plans, irrespective of the state’s benevolence towards its own population and its concrete commitment to results in health improvements for those most affected [32,33]. The idea of government accountability assumes the legitimacy of governments, while in absence of it, strengthening government systems might rather increase tensions among population groups, not to mention toward aid organizations implicated in such support [34]. Moreover, in contexts where (aid to) health care is an important source of resources, neglecting issues of political and vested interests linked to health systems can be conflictual per se and is likely to backfire.

In post conflict and post crisis situations, the question as to when (and how) to move from emergency to development remains a source of intense political debate. In practice, this judgment depends on context but also on political factors. Hence, who decides if conflict is over and if state building should be on the agenda, determines a major part of the process and access to potential resources. Under the New Deal initiative, judging the sufficient recovery of countries after conflict is left mainly to an evaluation by government and development actors [35]. It is unclear if and how humanitarian actors will be included; most humanitarian actors are not involved in the recent assessments in DRC, South Sudan, Sierra Leone and Liberia.

### Applying a ‘do no harm’ principle

Besides the possible absence of the desired effects [36] one cannot exclude unintended negative consequences in such volatile environments. Several donor policy papers include ‘do no harm’ guidance but these concentrate on potential undermining of the state building process [37,38]. We would argue there is also a place to anticipate, discuss and monitor potential negative effects of the current state building approach on health interventions and health results, including effects on humanitarian aid. Previous experience in contexts where development and humanitarian approaches co-exist, have revealed significant tensions, in particular around dominance of the political agenda over the humanitarian principles, with a risk of less effectiveness of humanitarian aid to those most in need.

We suggest at least three areas of concern: compromises in quality and effectiveness of humanitarian response; reduced health benefits as result of supported health interventions; changes in perception of health workers and interventions.

## Compromised humanitarian response under a health systems for state building approach

In the health systems approach according to the World Health organization [39] service delivery is only one of six building blocks, within a larger framework of wider, longer term objectives to strengthen systemic capacity. This can be at odds with goals more typically associated with immediate emergency response to populations’ needs, central to humanitarian action [1]. The adaptive capacity of health systems to increased or emerging health needs linked to crisis differs strongly from context to context, and may also advance and regress over time. In weak, deficient or inequitable health systems in particular, the timeframe needed for expected improvements is uncertain and in case of setbacks in the situation, e.g. renewed violence or increased direct health needs following outbreaks or influx of displaced people, it has regularly proven difficult to ‘change gears’. Health systems often fail to respond effectively to these renewed needs through accessible services with direct health impact, as described in Democratic Republic of Congo (DRC) [40] or Ivory Coast and Liberia [41].

In these situations, a significant tension is created between health interventions responding rapidly and effectively to urgent health needs of the most vulnerable and those with longer term aspirations of improving existing health systems. This can amount to barely concealed hostility, such as illustrated by a passage in the DRC’s national strategy on health systems strengthening, a policy most development actors in DRC have adopted. It states that humanitarian interventions had an […essentially ad hoc] approach, which should have given […way to action for development] and are described as [… entrenched, concealing their true nature: a tool disorganizing the health system in DRC] [42].

In violence and conflict affected contexts, humanitarian interventions may be also viewed as antagonists of system development and their benefit considered ‘out of place’ once the country is declared in a reconstruction and development phase. Especially because the distinction between conflict and post-conflict can be so imprecise, it is worth to note the highly political nature of determining when conflict or crisis ends. Many states show reluctance to accept the continued role of war, violence and socio-economic precariousness as part of the context reality, as illustrated in the country’s self-assessments under the New Deal. Often this recognition is feared to delay or make the transition to development less clear cut, or even could raise questions about the legitimacy of development-oriented arrangements that coincide with potential vested interests of the existing system and state institutions.

A typical and recurring tension in crisis situations concerns patient fees. The potential negative consequences of making patients pay for essential health care are well known [43]. In emergency contexts in particular the importance of accessing essential care without direct payment is recognised, including by major donors [44]. However, some development actors consider provision of care free of charge as depriving health systems of income from patient fees. Even if user fees’ contribution to sustained services is questionable and currently widely questioned [45], a systems perspective often outweighs the expected benefits for patients in terms of access, quality of care, potential impact and mitigation of iatrogenic poverty. In many weak and underfunded health systems patient fees are still considered as an acceptable ‘survival strategy’ of the system. MSF experience in DRC has shown reluctance by health facilities to declare outbreaks, because this would imply suspension of patient fees. Today, in DRC and Central African Republic (CAR), patients need to pay in order to obtain essential care, in spite of the widely recognised crisis.

The tension between rapid effectiveness and systems development appeared also in Haiti after the 2010 earthquake, with a [… national health system … still struggling to respond to the huge needs it continues to face] [46]. In particular for health actors with mixed mandates, the tension created by the collision of humanitarian and development approaches is reflected in practical operational choices. Downscaling of the health intervention relatively fast after the acute emergency phase is often felt as necessary on basis of concerns over sustainability of the current health care response and limitations of national capacity. Often it is implied that the primary intention of the emergency intervention should have at least included some measurable system benefit. This can lead to compromises in effective and timely humanitarian intervention, in spite of recognised continued needs of the population and continued weakness of the national health system to respond to these needs.

The paradox is that the weaker health systems are, the stronger the development impetus is to strengthen their capacity for the future, but also that re-establishing reliable health care delivery needed by the population within a reasonable timeframe through the existing weak(ened) system is quite a challenge. Still, in post crisis, investing in systems for better health outcomes in the long term proves often quite a gamble, as violence is often cyclical and even natural disasters show a clustering and repetitive tendency [7]. Hence, support of the state health system in so called ‘fragile states’ is less likely to translate into concrete health impact for the population, and in particular vulnerable groups might be/remain excluded due to pre-crisis inequity or shortfalls in the reconstructed system.

## Health systems support for state building potentially leading to less effective health response

Where health systems support aims to contribute to conflict transformation or state building, a possible shift can be expected towards outcomes other than treating patients and healthy populations. Its focus as regards the main priority of a health system – curing sick people and preventing disease- might be lost [21]. This might bring additional challenges to finding a healthy balance between desired outcomes and to keeping people’s health benefits central. A group of NGOs in South Sudan cited this concern in a recent statement [47]: “… political and financial support to the Government of South Sudan has, until now, been generally quite high, but support to the humanitarian needs of the people has sometimes wavered. Whilst recognizing the importance of building national institutions, the recent crisis has highlighted that a focus on ‘state building’ can come at the expense of supporting sustainable peace and development that *all* South Sudanese can benefit from.”

In practice, the current focus on health systems strengthening often sidelines delivery models complementing or parallel to the formal –often governmental- health care services. Arguably, in cases where state building is part of the wider objective, supporting and building credible health care institutions as part of the state could further take precedence over immediate and effective service delivery. Non-Governmental Organisations’ (NGO) interventions outside government coordination are seen as less desirable, not only for health systems but also for state legitimacy [5,34]. This is counterproductive, in particular in countries where large and effective medical networks linked to NGOs and faith based organisations provide a large proportion of health services, such as in Liberia, DRC, Haiti and elsewhere. If maximising impact through these non-governmental or faith-based networks seems a promising strategy, the state building agenda might nevertheless favor the use of the governmental health system at all costs. Recent examples from Islamic NGOs in the Middle East illustrate how different actors might find different ways to access people in need, depending on varying levels of cultural proximity or belonging in certain geographic areas [48]. In contrast, within the approach proposed by the New Deal, the preferential channeling of resources to country systems and towards national capacities is central: “International partners will increase the percentage of aid delivered through country systems on the basis of measures and targets jointly agreed at country level” [49].

## Changing perceptions of health interventions and actors

Harnessing health systems support for state building efforts raises questions on the implications of such an approach for humanitarian access, against the background of the long debate on “blurring the lines” between humanitarian action and other objectives, and its potential impact on the population’s perception of humanitarians [50,51], and thus on the safe access of patients to care and humanitarian access to patients. Embedding objectives of state building in health care provision might reinforce perceptions of partiality, including of humanitarian health actors.

The ever more prominent agendas of state-building and stabilization, as well as other “coherent” approaches to humanitarian aid, only further complicate the challenging endeavor of medical humanitarian work, putting humanitarian principles, operations and practitioners themselves at increased risk [4]. Documentation on politicization of humanitarian aid has advanced significantly in recent years. The next step will be to examine the field-level implications for humanitarian and other health interventions of the current approach proposed for fragile contexts with state-building and systemic capacity at its centre. Will over time any health intervention –including humanitarian ones - be perceived mainly as a tool for state reinforcement?

The G7+ statement [52] points to the specific need for “[e]ffective programs that protect and strengthen the most vulnerable and reach the most remote and inaccessible areas…” in health and other sectors of human and social development. However, it asserts that “[a]id must be distributed fairly across the country to reduce risks of conflict, and ensure social inclusion and a common national identity that is respected by international partners.” This concept of aid as conflict risk prevention stands in contradiction with principles central to humanitarian aid such as impartial provision of aid, targeting and proportionality of assistance with needs.

In cases where the health system itself is attacked, used as a battleground [53,54] or perceived as partisan in the conflict, additional barriers to equitable and effective delivery and access to care emerge. In conflict situations in particular, neither the health system nor the state is an impartial bystander. Health structures have been targeted in armed attacks, but also have been used as instruments to “win hearts and minds” by parties in combat or to buy votes [21,55,56]. From the perspective of state building and stabilization, it has been suggested to target health care to those who pose the greatest threat to peace, rather than those most in need, even if for a limited period of time [57].

Although initiatives such as the ‘New Deal’ and the Post 2015 development agenda are clearly part and parcel of development policy, there is no mention that humanitarian aid and emergency medical response constitute a distinct exception to the approaches proposed. Moreover, in practice, this distinction between development and humanitarian actors is likely to remain unclear in the perception of the general population and armed groups, with potentially negative consequences on access of medical humanitarian actors to populations and populations’ access to assistance [58].

With all that is at stake—compromises in quality and access to health care, aggravation of tensions over health (care) as a resource, the problem of unwilling governments—the burden of proof lies with this approach to show how it will achieve transformative objectives without compromising the lives and health of those in need of care in fragile settings.

## Summary

The current trend in global health discussions and health aid in particular to focus on ‘fragile and conflict affected states’ could further increase tensions into a highly volatile working environment. In particular, state and peace building objectives might transform the strategic choices, the modalities and perception of health responses for people in precarious health and living conditions.

In particular, the explicit focus on conflict and fragility within the aid effectiveness agenda and the post 2015 MDG discussions, might push health care for people affected by crisis and conflict to its limits. In these contexts, strategic choices geared towards health care support for patients and effective delivery models, might increasingly be influenced by the political framework of state ownership, state building and stabilization. Effective patient care and population based results might be sidelined or compromised by approaches that better fit potential transformative and systems aspects of health interventions. Ultimately, this approach could profoundly influence operational choices about type of interventions and approaches to targeting beneficiaries because of expected state building benefits, potentially reinforcing perceptions of health as partisan to or supporting parties in power.

We identified specific risks of these recent state-building initiatives for medical-humanitarian action and effective health delivery in conflict affected and fragile contexts. Significant tensions might grow around results expected from and obtained by health interventions. In particular, impartial and proportional aid, setting health benefits as priority, choices of most effective health strategies and targeting of the most vulnerable people might come under strain by striving to reinforce state stability and legitimacy as the overriding objective. Ultimately, there is a risk of missing out on existing health needs because it may not be politically feasible to point to vulnerabilities, gaps or moments where there are setbacks in the health system, as state capacity becomes the locus where progress is measured.

Further blurring of the different mandates and responsibilities might lead to decreased understanding and acceptance of humanitarian organisations, but also to questioning health workers’ genuine intention to privilege health services above political, ethnic or other alliances. In a global framework there might be a growing lack (or loss) of recognition that agencies with a humanitarian mandate do not necessarily support the wider state building agenda. This will further complicate risks for perception of aid personnel, the secure access of humanitarians to populations in need, and their access to assistance and medical care when needed.

In analogy to the well-developed debate about the distinct nature of humanitarian space (including politicization of aid and the tension or linking of humanitarian and development interventions), today there is an urgent need for debate about the distinct space for medical interventions in crisis situations, in order to be able to obtain the full, optimal impact of health interventions in response to urgent medical needs. This discussion should include the expected health outcomes for populations in crisis and how to preserve these within a wider policy agenda currently shifting towards state building and resilient health systems. In particular, caution is in order where aid is confronted with conflict or violence, the unwillingness of governments and/or vested interests and competition for scarce resources. From a medical-humanitarian perspective, it is crucial to keep direct health and humanitarian results for the population at the centre of post crisis interventions and not compromise on this concrete and immediate objective, even if deemed less interesting for state building or other political aspirations.

At present, the lack of concrete evidence on feasibility and benefits of health care for state building seems to hardly reduce the enthusiasm for it as a political project. When such evidence does emerge, this should not lead to a backlash towards less humanitarian aid or less aid for health. First and foremost, health and humanitarian investments must be gauged on the basis of how they can improve people’s lives and wellbeing-- and this approach, based on long-standing best practices, cannot be compromised without significant human impact. Given what is at stake in situations of crisis, conflict or fragility in particular, effective health and humanitarian interventions in response to people’s immediate needs can make all the difference and should not be undermined by today’s aspirations towards state building and the wider transformative project of health systems support.

## Abbreviations

AIDS: Acquired Immuno Deficiency Syndrome
CAR: Central African Republic
DRC: Democratic Republic of Congo
FCAS: Fragile and Conflict Affected States
GAVI: Global Alliance for Vaccines and Immunisations
GFATM: Global Fund for AIDS, Tuberculosis and Malaria
MDG: Millennium Development Goals
MSF: Médecins Sans Frontières
NGO: Non governmental organization
PEPFAR: President’s Emergency Plan For Aids Relief
WHO: World Health Organisation
